# The effects of response time on older and young adults’ interaction experience with Chatbot

**DOI:** 10.1186/s40359-025-02459-9

**Published:** 2025-02-21

**Authors:** Ya-Ling Wang, Chi-Wen Lo

**Affiliations:** National Taiwan Normal University, Taipei, Taiwan

**Keywords:** Age difference, Chatbots, Delayed response, Older adults, Response time

## Abstract

**Background:**

Chatbots, such as Siri, Alexa, and ChatGPT, are increasingly integrated into various domains, including customer service and virtual companionship, transforming human-computer interactions. However, there remains limited understanding of how response time—a critical social cue—affects user experience across different age groups, particularly in virtual companionship contexts. This gap is especially relevant in aging societies where older adults’ emotional and relational needs require tailored technological solutions.

**Methods:**

A 2 × 2 between-subjects experiment was conducted with 160 Taiwanese participants from two age groups: young adults (18–23 years old) and older adults (56–81 years old). Participants interacted with chatbots under two conditions: instant responses (approximately 3 s) and delayed responses (10–60 s). User experience was measured using social presence (SP), service encounter satisfaction (SAT), and intention of use (IOU) through validated questionnaires. Two-way ANOVA was employed to analyze the main and interaction effects of response time and age group.

**Results:**

The findings revealed a significant interaction effect between response time and age group. While younger adults preferred instant responses, older adults showed a preference for delayed responses. Specifically: (1) Instant responses led to higher satisfaction and engagement for younger adults, who value efficiency and immediacy. (2) Delayed responses facilitated cognitive comfort and enhanced relational value for older adults, emphasizing the importance of conversational pacing.

**Conclusions:**

This study highlights the critical role of response time in chatbot design, revealing how age-specific preferences influence user satisfaction in virtual companionship scenarios. Findings underscore the need for adaptive chatbot designs that align with cognitive and emotional needs across age groups. Broader implications emphasize the importance of balancing digitally assisted companionship with the risks of dehumanization. Future research should explore long-term interaction effects and cultural differences to enhance chatbot inclusivity and effectiveness.

## Introduction

The rapid growth of the aging population in Taiwan has brought significant societal challenges. In 2018, Taiwan officially entered the United Nations-defined aging society, with the elderly population (65 years and older) accounting for 14% of the total population. By 2025, this figure is projected to reach 20%, classifying Taiwan as a super-aged society [1]. This demographic shift emphasizes the need to address not only older adults’ physical health but also their emotional well-being. Emotional companionship is crucial for maintaining mental health and quality of life in this population. Recognizing this, the Taiwanese government has initiated programs to develop companion robots that provide emotional support [2].

Chatbots, as conversational agents, hold potential as accessible tools to fulfill these companionship needs. However, the effectiveness of chatbots relies heavily on their ability to simulate natural interactions, with response time being a particularly important factor. Tailored response time has been shown to significantly influence user experience, particularly in enhancing life quality for older adults [3]. Yet, how response time impacts user satisfaction across different age groups remains unclear, especially in virtual companionship contexts.

While younger adults typically value fast-paced, efficient interactions with chatbots, older adults may prefer conversational pacing that aligns with their cognitive and emotional processing. This divergence underscores a critical gap in understanding how chatbot response time affects user experience for different age groups. Addressing this gap is vital for designing inclusive and effective chatbots that cater to diverse user needs.

This study aims to explore the impact of chatbot response time on user experiences, focusing on differences between older and younger adults in a virtual companionship context. By examining these variations, we seek to provide insights into tailoring chatbot designs to meet the unique needs of an aging population.

## Response time of Chatbot

### Chatbots and social cues

Chatbots, or conversational agents, are software applications designed to simulate human-like interactions through text or voice-based interfaces. According to Dale [4], chatbots engage users via conversational interfaces, enabling natural language communication and information exchange. These systems are widely used in customer service, education, healthcare, and companionship contexts, owing to their scalability and efficiency in facilitating user interactions [5, 6].

Chatbots can be broadly categorized into three types based on their scope and functionality [6]:

- Virtual Companions: Designed for deep and sustained interactions, these chatbots provide companionship and emotional support, often catering to populations like older adults who seek relational and meaningful engagement.
- Intelligent Assistants: Examples include Siri, Alexa, and Google Assistant, which perform a wide variety of tasks but typically engage users in brief, goal-oriented interactions.
- Task-Focused Chatbots: These chatbots are specialized for specific tasks, such as customer service inquiries, and operate with narrow but deep functionality.

This study focuses on virtual companions, which are particularly relevant for addressing the relational and emotional needs of older adults. These chatbots prioritize creating meaningful, human-like interactions over task completion or transactional efficiency.

Social cues, such as response timing, play a crucial role in shaping user perceptions of chatbot interactions. Social Response Theory (SRT) [7], proposed by Nass and Moon, explains how humans instinctively apply social norms when interacting with computers and other technologies that exhibit human-like characteristics. SRT has been widely adapted to human-chatbot interactions, demonstrating that even minimal cues, such as language style, tone, and response timing, are sufficient to trigger social responses [8–10]. Response timing, in particular, is critical in establishing perceptions of humanness, trustworthiness, and social presence, even in the absence of visual or auditory human-like features [10–12].

Building on SRT, Anthropomorphism Theory provides a broader framework for understanding why users attribute human-like characteristics to chatbots. The theory posits that humans anthropomorphize technologies to make sense of their interactions, especially when the technology exhibits behaviors (e.g., timely responses) that align with human social norms [13]. In this context, response timing functions as a proxy for human intentionality and emotional engagement, influencing perceptions of the chatbot’s warmth and relational value [14]. For example, moderate response delays can foster a sense of conversational naturalness, enhancing user perceptions of the chatbot’s human-like qualities. Conversely, overly fast or excessively delayed responses disrupt these perceptions, diminishing engagement and satisfaction [12].

Although SRT traditionally emphasizes multimodal social cues like facial expressions and gestures, its relevance to chatbot interactions without these features is supported by studies highlighting the importance of timing and responsiveness in text-based contexts. Reeves and Nass [15] emphasized that even in the absence of physical human-like traits, timing alone can significantly influence user perceptions of engagement and humanness. These findings align with Anthropomorphism Theory, which suggests that response timing triggers social expectations [13].

### Importance of response time

Response time is a critical social cue in chatbot interactions, shaping user satisfaction, conversational flow, and relational engagement [16–21]. Its influence varies depending on the interaction context, user expectations, and demographic characteristics. In customer service scenarios, for example, users typically expect rapid responses for efficiency and task completion [18, 22]. In contrast, virtual companionship prioritizes conversational pacing that fosters emotional connection and trust, where moderate delays may enhance the perception of human-likeness and social presence [10, 12].

The evaluation of response time is closely tied to both the context of interaction and the characteristics of user groups. Older adults may prefer delayed responses that align with their cognitive processing speeds [23] and their focus on meaningful, emotionally satisfying exchanges [24, 25]. Younger adults, on the other hand, often value instant responses due to their familiarity with fast-paced digital communication and their preference for efficiency [26, 27] By examining how response time influences user perceptions across contexts and demographics, this study addresses key gaps in the existing literature, particularly in virtual companionship scenarios.

#### The dual effects of response time across contexts

##### Positive influences

Moderate response delays often enhance conversational naturalness, social presence, and perceptions of humanness. For example, Moon [10] found that appropriately delayed responses increased source credibility and information quality in virtual companion chatbots, fostering stronger relational bonds. Similarly, Gnewuch et al. [12] demonstrated that moderate delays create a sense of natural conversational pacing, improving user satisfaction in customer service interactions.

In companionship contexts, delayed responses are particularly beneficial, as they mimic human conversational rhythms, fostering trust and emotional engagement [28, 29]. Timing delays of approximately one second have been identified as optimal, balancing human-like relatability and efficiency [30]. These findings suggest that moderate delays enhance the relational quality of chatbot interactions, particularly in contexts where empathy and connection are essential.

##### Negative influences

Conversely, excessive delays often disrupt conversational flow and reduce user satisfaction. Holtgraves et al. [31] observed that instant responses were associated with higher ratings of extraversion and responsiveness, while longer delays led to disengagement. In task-oriented or emotionally charged scenarios, delays exceeding user expectations can result in frustration and diminished trust [32, 33].

For example, excessively long delays in customer service or companionship scenarios may create an impression of inefficiency, leading to dissatisfaction and reduced engagement. These findings highlight the need for careful calibration of response timing to align with user expectations and context-specific requirements. In companionship scenarios, while moderate delays can enhance the interaction by fostering naturalness and empathy, excessively long delays may compromise relational engagement and reduce trust.

## Factors influencing Interaction experiences with Chatbots

Interaction experiences with chatbots are shaped by a combination of technological factors and demographic characteristics, which influence user satisfaction, trust, and engagement. Categorizing these factors provides insights into how chatbot designs can be tailored to diverse user needs.

### Technological factors

The following technological factors significantly affect user experiences with chatbots:

#### Response time

Response time is a critical social cue that impacts conversational flow, trust, and satisfaction. Moderate delays improve conversational naturalness and perceptions of humanness, especially in companionship contexts. However, excessive delays disrupt flow and diminish satisfaction [10, 12].

#### Usability and simplicity

Chatbots with clear, intuitive designs improve user engagement and reduce frustration. Older adults, in particular, value simplicity and usability, as they often face challenges with unfamiliar technology or technophobia [34, 35].

#### Trustworthiness and security

Trust is a key determinant of chatbot adoption and engagement, especially among older adults who are more concerned about privacy and security. Transparent and reliable chatbot performance builds trust for all user groups [36].

### Demographic factors

Demographic factors, particularly age, strongly influence chatbot interaction preferences. These differences highlight the need for customized designs to cater to diverse user groups:

#### Younger adults

Younger users, accustomed to fast-paced digital environments, expect efficient and immediate interactions [37, 38]. Their preferences are explained by:

- Arousal Theory, which links quick responses to heightened emotional engagement [26, 27].
- Expectancy-Confirmation Theory, where meeting expectations for immediacy reinforces trust and satisfaction [39, 40].

#### Older adults

Older users prefer slower, deliberate interactions that align with their cognitive and emotional needs. These preferences are supported by:

- Processing-Speed Theory, which highlights that slower cognitive processing makes delayed responses more comfortable [23].
- Socioemotional Selectivity Theory, which emphasizes older adults’ focus on meaningful, emotionally satisfying interactions [24, 25].

Van der Goot and Pilgrim [34] found that older adults prioritize simplicity and trustworthiness, while Gudala et al. [36] demonstrated that well-designed chatbots improve medication adherence among older users. These insights underscore the need for designs that align with each demographic’s expectations. For instance, delayed responses may enhance older adults’ experiences by supporting their socioemotional goals, while instant responses better meet younger adults’ demands for efficiency and immediacy.

## Overview of the study

This study investigates how chatbot response time impacts user interaction experiences, particularly in virtual companionship contexts, with a focus on differences between older and younger adults. The research is motivated by the need to design age-inclusive chatbots that address the emotional and relational needs of an aging population while meeting the efficiency expectations of younger users.

### Core research question

How does chatbot response time (instant vs. delayed) influence user interaction experiences across different age groups, particularly in virtual companionship scenarios?

### Rationale

The rapid growth of aging societies globally necessitates technologies that can provide emotional and relational support to older adults. Chatbots, as virtual companions, have the potential to fulfill these needs. However, response time, a critical social cue, has been understudied in the context of virtual companionship. Addressing this gap is essential for designing chatbots that cater to the cognitive and emotional preferences of diverse age groups.

### Objectives

This study aims to address the following objectives:

1. Examine how chatbot response time (instant vs. delayed) affects user experience in virtual companionship contexts.
2. Investigate differences in how older and younger adults perceive and evaluate chatbot interactions.
3. Explore the interaction effects of response time and age group on user satisfaction, informed by psychological theories such as:
4. Processing-speed theory and socioemotional selectivity theory to explain older adults’ preferences for delayed responses [27, 28].
5. Arousal theory and expectancy-confirmation theory to understand younger adults’ preference for instant responses [29, 30].

These objectives and theoretical underpinnings form the foundation of the study, guiding its design and analysis to provide actionable insights for the development of adaptive chatbot systems.

## Method

### Participants

The current study recruited a total of 160 participants in Taiwan using convenience sampling. The participants were divided into two age groups:

1. Young Adults: *N* = 77 (62 females, 15 males), aged 18–23 years (M = 19.12, SD = 0.95).
2. Older Adults: *N* = 83 (57 females, 26 males), aged 56–81 years (M = 68.42, SD = 5.01).

Young adults were recruited from university campuses, while older adults were recruited from social educational institutions. Table 1 provides demographic details, including education level, retirement status, and marital status. The age criterion for older adults aligns with the United Nations’ definition of individuals aged 60 and above, though the range was expanded slightly to include participants nearing this threshold.

**Table 1:** Demographics of participants

		Older Adults (*N* = 83)	Young Adults (*N* = 77)	Total (*N* = 160)
**Gender**	male	26 (31.33%)	15 (19.48%)	41 (25.63%)
	female	57 (68.67%)	62 (80.52%)	119 (74.38%)
**Education level**	elementary education	2 (2.41%)	-	2 (1.25%)
	junior high school education	5 (6.02%)	-	6 (3.13%)
	high school education	19 (22.89%)	-	19 (11.88%)
	college degree	46 (55.42%)	77 (100%)	123 (76.88%)
	graduate degree or above	9 (10.84%)	-	9 (5.63%)
	did not report	2 (2.41%)	-	2 (1.25%)
**Retirement status**	retired	75 (90.36%)	-	75 (46.88%)
	still working	6 (7.23%)	77 (100%)	83 (51.88%)
	did not report	2 (2.41%)	-	2 (1.25%)
**Marital status**	unmarried	7 (8.43%)	77 (100%)	84 (52.5%)
	married and living together	57 (68.67%)	-	57 (35.63%)
	Divorced or not living together	6 (7.23%)	-	6 (3.75%)
	widowed	11 (13.25%)	-	11 (6.88%)
	did not specify	2 (2.41%)	-	2 (1.25%)

### Experimental design

This study employed a 2 × 2 between-subjects design to examine the effects of age group (young vs. older adults) and response time (instant vs. delayed responses) on user experience. Participants were randomly assigned to one of four experimental conditions:

1. Young Adults × Instant Responses (*N* = 30).
2. Young Adults × Delayed Responses (*N* = 47).
3. Older Adults × Instant Responses (*N* = 47).
4. Older Adults × Delayed Responses (*N* = 36).

#### Response time conditions

1. *Instant Responses*: Chatbot replies were provided within 0.1 s, simulating real-time interaction.
2. *Delayed Responses*: Chatbot replies exhibited delays randomly ranging from 10 to 60 s, mimicking human conversational pacing with variability.

Participants interacted with the chatbot over a 5-day period, engaging in at least one meaningful conversation per day. On average, participants spent 15–20 min per day interacting with the chatbot, totaling approximately 75–100 min over the study period. This duration was chosen to capture short-term user impressions while preventing fatigue. Specific interaction time data was monitored and recorded to ensure consistent engagement across groups.

Following the 5-day interaction period, participants completed a questionnaire evaluating their experiences. Measures included social presence (SP), service encounter satisfaction (SAT), and intention of use (IOU), each designed to assess different aspects of user interaction.

### Procedure

The study consisted of three sessions: an orientation session, a 5-day interaction session, and a questionnaire and debriefing session.

#### Session 1: orientation session

Participants were introduced to the chatbot and tasks, provided informed consent, and submitted demographic information (age, gender, education level).

#### Session 2: interaction session

Participants engaged in meaningful conversations with the chatbot daily for 5 days. A “meaningful conversation” was defined as exchanges exhibiting coherence and logical flow. The chatbot sent reminder messages three times a day (9 a.m., 2 p.m., and 7 p.m.) to encourage participation. The number of daily messages sent by participants was recorded to ensure sufficient engagement.

#### Session 3: questionnaire and debriefing session

Participants completed a post-interaction questionnaire evaluating their perceptions of the chatbot and their overall experience. A debriefing session followed, during which participants were thanked and compensated with 300 NT dollars.

### Instruments

#### Chatbot

The chatbot used in this study was developed through the LINE messaging application, powered by GPT-3 (text-davinci-001). It featured four distinct dialogue agents, each designed with unique personality traits to simulate diverse conversational dynamics:

* Jenny*: Warm and kind.

* Anne*: Negative.

* Jack*: Optimistic and enthusiastic.

* Tom*: Conservative and emotionally stable.

Participants received responses from all four agents for each message they sent. The responses were generated using carefully crafted prompts to ensure consistency in personality expression across agents. To avoid systematic bias, the order of responses from the agents was randomized for each participant, ensuring balanced exposure to all four personalities throughout the study.

Although no formal pretest was conducted to evaluate individual agent effects, the randomization strategy and the study’s primary focus on response-time effects helped mitigate potential confounding influences arising from agent-specific characteristics.

### Measures

The questionnaire assessed social presence (SP), service encounter satisfaction (SAT), and intention of use (IOU), adapted from established scales. Items were rated on a 7-point Likert scale (1 = “strongly disagree”; 7 = “strongly agree”).

* Social Presence*: 5 items (composite reliability = 0.95).

* Service encounter satisfaction*: 3 items (composite reliability = 0.90).

* Intention Of Use*: 3 items (Cronbach’s α = 0.98).

### Data analysis

Data analysis was conducted using SPSS version 23 and involved:

1. Descriptive analysis of demographic characteristics.
2. Preliminary analysis of SP, SAT, and IOU measures, including reliability, validity, and correlations.
3. Two-way ANOVA to examine the effects of age group and response time on SP, SAT, and IOU.

## Results

### Preliminary analyses

According to the variables of this study, including SP, SAT and IOU, the descriptive statistical analysis including min, max, average score and standard deviation, reliability, validity and correlation are shown in Table 2. Table 3 shows the average score and standard deviation calculated by group. All variables have good reliability and validity and are all significantly correlated.

**Table 2:** Descriptivestatisticallanalysiss,reliabilityy andvalidityy of measuress

								Discriminant Validity & Correlation Coefficient		
	Min	Max	Mean	SD	**α**	CR	AVE	SP	SAT	IOU
SP	1.4	7	4.24	1.11	0.79	0.76	0.44	(0.66)		
SAT	1	7	3.83	1.27	0.88	0.83	0.63	0.73^**^	(0.79)	
IOU	1	7	4.25	1.50	0.95	0.89	0.72	0.69^**^	0.72^**^	(0.85)

Note: SP, social presence; SAT, satisfaction; IOU, intention of use; SD, standard deviation; **α**, Cronbach’s alpha; CR, composite reliability; AVE, average variance extracted; ( ), square root of AVE; ^**^p <.01

**Table 3:** Descriptive statistical analysis of measures (by Group)

	*N*			SP M (SD)			SAT M (SD)			IOU M (SD)			
	instant	delayed	Total	instant	delayed	Total	instant	delayed	Total	instant	delayed	Total	
Older adult	47	36	83	4.46(1.05)	4.65(1.16)	4.54(1.10)	3.89(1.16)	4.20(1.51)	4.02(1.32)	4.73(1.23)	4.64(1.56)	4.69(1.38)	
Young adult	30	47	77	4.06(0.90)	3.83(1.11)	3.92(1.03)	4.11(1.12)	3.29(1.13)	3.61(1.19)	4.33(1.27)	3.43(1.52)	3.78(1.49)	
Total	77	83	160	4.30(1.01)	4.19(1.20)	4.24(1.11)	3.97(1.14)	3.69(1.37)	3.83(1.27)	4.58(1.26)	3.95(1.64)	4.25(1.50)	

Note: SP, social presence; SAT, satisfaction; IOU, intention of use; M, mean; SD, standard deviation

### Two-way ANOVA

The significance of older adults’ and young adults’ SAT, SP and IOU changes according to chatbot’s response time was determined with two-way ANOVA. The results obtained from the test were given in Table 4.

**Table 4:** ANOVA analysis

		SS	df	MS	F
SP	Age	14.15	1	14.15	12.40^**^
	Response Time	0.01	1	0.01	0.01
	Age * Response Time	1.71	1	1.71	1.50
	Error	178.12	156	1.14	
SAT	Age	4.57	1	4.57	3.02^†^
	Response Time	2.44	1	2.44	1.62
	Age * Response Time	12.48	1	12.48	8.26^**^
	Error	235.89	156	1.51	
IOU	Age	25.02	1	25.02	12.69^**^
	Response Time	9.64	1	9.64	4.89^*^
	Age * Response Time	6.43	1	6.43	3.26^†^
	Error	307.49	156	1.97	

Note: SP, social presence; SAT, satisfaction; IOU, intention of use; *SS*, sum of square; *df*, degrees of freedom; *MS*, mean of square; ^†^*p* <.10; ^*^p <.05; ^**^p <.01

#### Main effects

As shown in Table 3, chatbot’s response time had no significant main effects on SP (*F* = 0.01, *p* >.05) and SAT (*F* = 1.62, *p* >.05). However, a significant main effect was observed for IOU (*F* = 4.89, *p* <.05), where IOU scores for instant responses (*M* = 4.58, *SD* = 1.26) were greater than for delayed responses (*M* = 3.95, *SD* = 1.64).Significant main effects of age group were observed for SP (*F* = 12.40, *p* <.001) and IOU (*F* = 12.69, *p* <.001), and a marginally significant effect for SAT (*F* = 13.02, *p* =.058). Older adults scored higher than young adults across all three measures: SP for older adults (*M* = 4.54, *SD* = 1.10) exceeded that of young adults (*M* = 3.92, *SD* = 1.03); IOU for older adults (*M* = 4.69, *SD* = 1.38) was greater than for young adults (*M* = 3.78, *SD* = 1.49); and SAT for older adults (*M* = 4.02, *SD* = 1.32) exceeded that of young adults (*M* = 3.61, *SD* = 1.19).

#### Interaction effects

In addition to main effects, Table 4 notes significant interaction effects between age group and response time on SAT (*F* = 8.26, *p* <.01) and IOU (*F* = 3.26, *p* =.075). These interactions indicate that the impact of response time on user experience differs between older and younger adults.

**Fig. 1:**
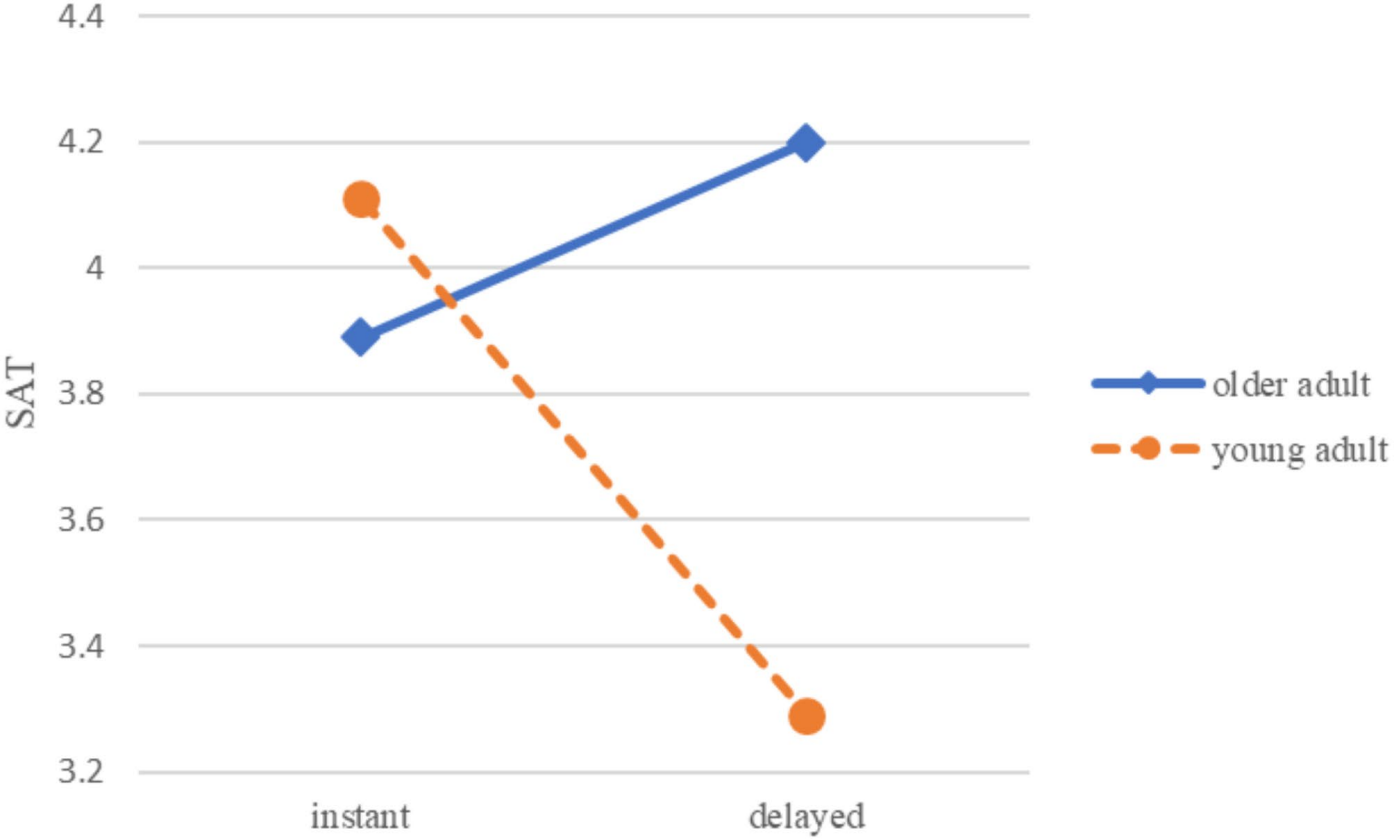
The interaction of response time and age on SAT

As illustrated in Fig. 1, for SAT, young adults scored higher when interacting with instant-response chatbots (*M* = 4.11, *SD* = 1.12) compared to older adults (*M* = 3.89, *SD* = 1.16). Conversely, for delayed-response chatbots, older adults scored higher (*M* = 4.20, *SD* = 1.51) than young adults (*M* = 3.29, *SD* = 1.13).

**Fig. 2:**
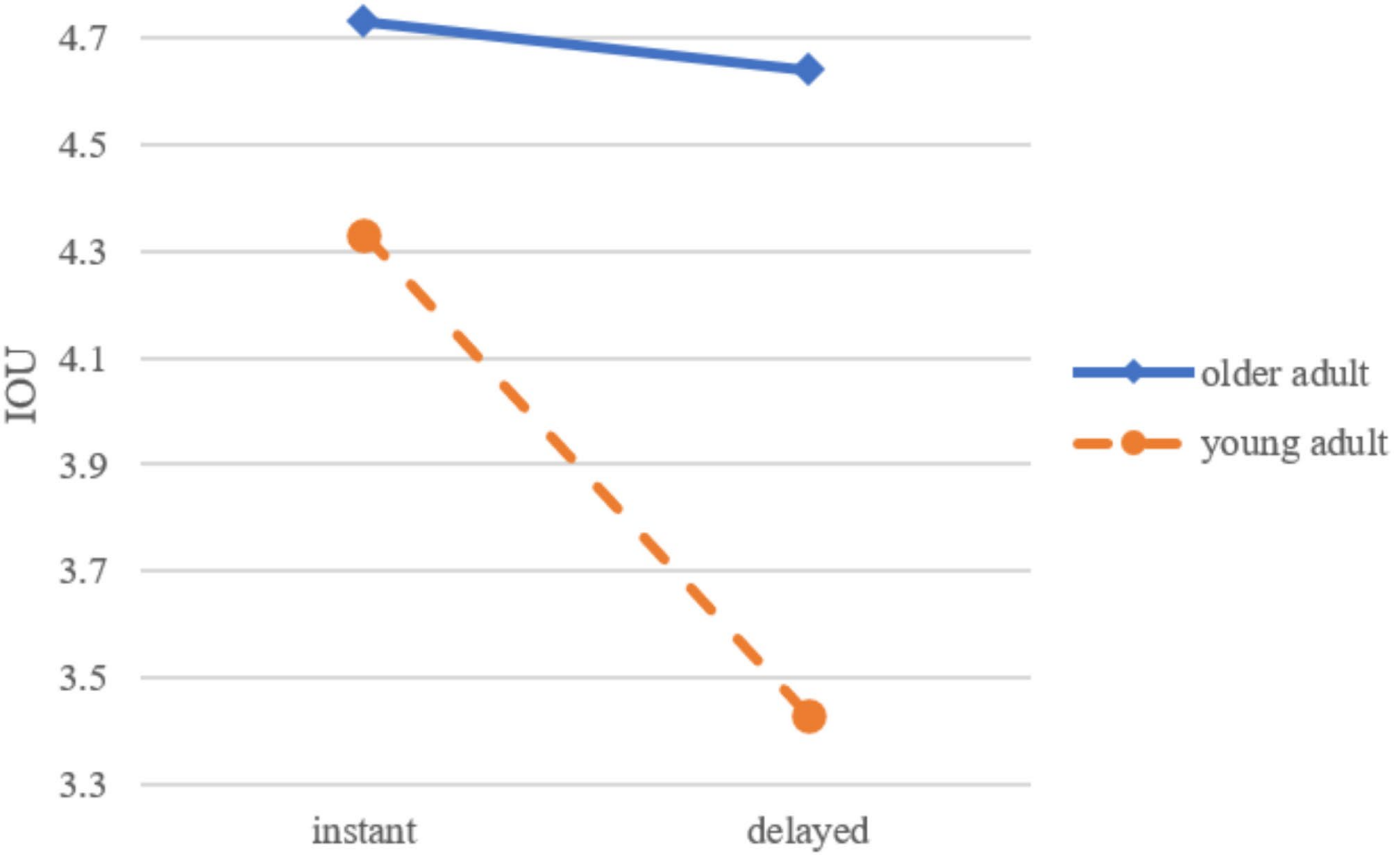
The interaction of response time and age on IOU

For IOU, as shown in Fig. 2, older adults (*M* = 4.73, *SD* = 1.23) and young adults (*M* = 4.33, *SD* = 1.27) had similar scores when interacting with instant-response chatbots. However, with delayed-response chatbots, IOU for older adults (*M* = 4.64, *SD* = 1.56) was notably higher than for young adults (*M* = 3.43, *SD* = 1.52).

These findings confirm that response time impacts were analyzed independently of age-related differences. While age groups displayed distinct preferences, significant main effects and interaction effects demonstrate that response time (instant vs. delayed) has a substantial and context-dependent impact on SP, IOU, and SAT. Interaction effects, in particular, highlight nuanced variations in how response time preferences manifest across age groups, further supporting the need for demographic-specific chatbot designs.

## Discussion

This study demonstrates that chatbot response time significantly impacts user interaction experiences, with distinct preferences between young and older adults. The findings, supported by psychological theories, provide valuable insights for tailoring chatbot designs to different user demographics. Below, we integrate psychological frameworks to discuss the four key findings:

### Impact of response time on intention of use (IOU)

Immediate responses led to more favorable IOU scores, as delays in instant messaging have been shown to reduce conversational involvement, increase frustration, and decrease user satisfaction [41]. This finding aligns with cognitive load theory, which posits that disruptions, such as delays, can increase cognitive demands, thereby reducing the fluidity of interactions and diminishing satisfaction [18, 19]. Furthermore, instant responses align with users’ expectations for immediacy, as explained by the expectancy-confirmation theory. When users’ expectations for quick and seamless communication are met, their perceptions of the chatbot’s performance and intention to continue use improve [39, 40].

In the context of virtual companionship, timely responses may enhance user engagement by fostering conversational flow and meeting emotional expectations. While our findings indicate that intention of use was significantly higher for instant responses, the observed trends in social presence (SP) and service encounter satisfaction (SAT) suggest that response timing may also influence relational and emotional engagement. These broader implications are supported by prior research but require further empirical validation. These findings emphasize the importance of calibrating response timing to match user needs, particularly for younger adults who value immediacy and efficiency in their interactions.

### Age differences in user interaction preferences

Older adults rated their chatbot experiences more positively than younger adults, regardless of response time. This finding aligns with socioemotional selectivity theory [24, 25], which posits that older adults prioritize emotionally meaningful interactions over technical precision as they focus on maximizing emotional satisfaction in social contexts. Their higher SAT and IOU scores reflect this tendency to view chatbots as valuable tools for communication and companionship, focusing on relational utility rather than performance.

In contrast, younger adults, who are more familiar with advanced technology, may have higher expectations for seamlessness and efficiency. According to the technology acceptance model (TAM) [42], users’ satisfaction is influenced by perceived ease of use and usefulness. Younger adults’ more critical assessments suggest that when chatbots fail to meet their high standards for speed and accuracy, dissatisfaction increases. These contrasting evaluations between older and younger adults underscore the importance of considering demographic differences when designing chatbots to enhance user satisfaction across age groups [43].

### Interaction effects of age and response time

This study revealed significant interaction effects between age and response time on user experience measures, particularly SAT (service encounter satisfaction) and IOU (intention of use). These findings indicate that age-related differences significantly influence how response time impacts user satisfaction and engagement, providing nuanced insights into designing chatbots tailored to diverse demographics.

For older adults, delayed responses were generally preferred. This preference aligns with processing-speed theory [23], which explains that age-related cognitive changes reduce the speed at which older individuals process information. Delayed responses allow sufficient time for comprehension, reducing cognitive overload and creating a more comfortable interaction. Additionally, parasocial interaction theory [44, 45] suggests that human-like conversational rhythms, achieved through delayed responses, foster a sense of companionship and relatability, particularly in virtual companionship contexts. The findings further align with socioemotional selectivity theory [24, 25], as older adults tend to prioritize emotionally meaningful and satisfying interactions over immediacy. Their higher SAT and IOU scores for delayed-response chatbots reflect this preference, highlighting the relational and emotional benefits they derive from slower-paced interactions [34].

By contrast, younger adults showed a clear preference for instant responses. Their higher SAT scores for instant-response chatbots reflect expectations for rapid communication, consistent with arousal theory [26, 27], which links quick feedback to heightened emotional engagement and satisfaction.

Moreover, expectancy-confirmation theory [39, 40] explains that younger users’ satisfaction is reinforced when chatbots meet their high expectations for immediacy and seamlessness. When response times were delayed, younger adults’ engagement and satisfaction significantly decreased, underscoring the importance of immediacy for maintaining a positive user experience in this demographic.

These interaction effects highlight the importance of context-sensitive chatbot design. For older adults, delayed responses enhance their interaction experience by aligning with cognitive and emotional needs. For younger adults, instant responses are critical to meet their expectations for speed and efficiency. Together, these findings emphasize the need to tailor chatbot response time to the specific preferences of diverse user groups to optimize engagement and satisfaction.

### Broader implications and future research

The findings of this study highlight the dual role of chatbots in fostering digitally assisted relationships while mitigating the risks of dehumanization. We expanded on the ethical considerations of using chatbots for companionship, emphasizing the importance of designing chatbots that complement rather than replace human relationships. Chatbots, when designed thoughtfully, can enhance accessibility and provide emotional support without replacing meaningful human connections. For instance, tools like XiaoIce demonstrate how empathetic chatbots can complement users’ social lives by fostering emotional connections while encouraging real-world interactions [46]. Future chatbot designs should focus on integrating features that encourage real-world social connections, such as promoting engagement with family or community activities, to strike a balance between digital support and human interaction [47]. Such measures can help mitigate the risks of dehumanization by positioning chatbots as supportive tools rather than substitutes for authentic human interactions.

Additionally, the 5-day experiment provided critical insights into short-term user perceptions and behavioral intentions. While this approach effectively captured early-stage user behavior, it also highlights the need for longitudinal research to explore how sustained chatbot interactions impact user preferences, engagement, and satisfaction over time. Prior research underscores the importance of extended interaction periods; for example, Schweitzer et al. [48] suggest that periods of three weeks or longer are essential to fully understand the relational dynamics of virtual companionship. Future studies should adopt longitudinal designs and naturalistic contexts to evaluate how chatbots fulfill relational and emotional needs in diverse scenarios.

## Limitations

While this study provides important insights into the effects of chatbot response time on user experience, certain limitations should be acknowledged. First, the 5-day interaction period was designed to capture short-term user impressions and initial behavioral intentions. This relatively brief duration may limit the generalizability of our findings to long-term use scenarios, where relationships with chatbots are likely to develop differently. Future studies could extend the duration of chatbot interactions to several weeks or months, as recommended in prior research [48], to gain a deeper understanding of how sustained interactions influence user perceptions and behavioral outcomes.

Additionally, while participants engaged in meaningful conversations daily for 15–20 min on average, the scope of these interactions may not fully replicate real-world use patterns where chatbot interactions occur sporadically or in specific contexts. Expanding the range of interaction scenarios and measuring cumulative engagement over a longer period could further enhance the ecological validity of the results.

Another limitation of this study is its generalizability across broader populations. While our sample size of 160 participants meets established standards for statistical validity in experimental research, we acknowledge that future studies could benefit from even larger and more diverse samples to confirm and extend our findings. Specifically, our study focused on a specific demographic in Taiwan, and preferences or behaviors of older and younger adults may differ across cultural or geographical contexts. Future research should consider recruiting participants from various cultural or geographical backgrounds to enhance the applicability of results.

Additionally, while the current study included a sufficient number of participants for each experimental group, there was a higher proportion of female participants. Future studies should aim for more gender-balanced samples to provide a more comprehensive understanding of chatbot interactions across diverse demographic characteristics. This approach will help uncover potential gender-related differences in user experiences and preferences.

## Conclusion

In conclusion, our study provides valuable insights into how different age groups perceive and interact with chatbots, particularly focusing on their preferences regarding response times. We found that young adults exhibit a clear preference for instant responses, aligning with their familiarity with fast-paced digital communication and expectations for immediate information access. In contrast, older adults demonstrated a preference for delayed responses, which may align better with their cognitive processing speeds and comfort with technology. These findings underscore the importance of considering demographic differences when designing and implementing chatbots to enhance user satisfaction and engagement across diverse user groups.

## Data Availability

The dataset of the present study is available upon request from the corresponding author (Ya-Ling Wang, email: ylwang47@gapps.ntnu.edu.tw).
